# Phylogenetic Insights into Chinese *Rubus* (Rosaceae) from Multiple Chloroplast and Nuclear DNAs

**DOI:** 10.3389/fpls.2016.00968

**Published:** 2016-06-29

**Authors:** Yan Wang, Qing Chen, Tao Chen, Haoru Tang, Lin Liu, Xiaorong Wang

**Affiliations:** ^1^College of Horticulture, Sichuan Agricultural UniversityChengdu, China; ^2^Institute of Pomology and Olericulture, Sichuan Agricultural UniversityChengdu, China; ^3^Agricultural and Animal Husbandry College of Tibet UniversityLinzhi, China

**Keywords:** Chinese *Rubus*, phylogeny, section *Idaeobatus*, section *Malachobatus*, hybridization, polyploidy

## Abstract

*Rubus* L. is a large and taxonomically complex genus, species of which exhibit apomixis, polyploidy, and frequent hybridization. Most of Chinese *Rubus* are assigned in two major sections, *Idaeobatus* and *Malachobatus*. To explore the phylogenetic relationships within Chinese *Rubus*, inferences upon three chloroplast DNA (*rbc*L, *rpl*20-*rps*12, and *trn*G-*trn*S), nuclear ribosomal ITS, and two low-copy nuclear markers (*GBSS*I-2 and *PEPC*) were deduced in 142 *Rubus* taxa from 17 subsections in 6 sections. *nrITS* and *GBSS*I-2 were the most informative among the six DNA regions assessed. Phylogenetic relationships within *Rubus* were well-resolved by combined nuclear datasets rather than chloroplast markers. The phylogenetic inferences strongly supported that section *Idaeobatus* was a polyphyletic group with four distant clades. All samples of sect. *Malachobatus* formed a monophyletic clade, in which *R. tsangorum* and *R. amphidasys* of sect. *Dalibardastrum*, and *R. peltatus* from subsection *Peltati* of sect. *Idaeobatus* were always nested. *Rubus pentagonus* (2*n* = 2*x* = 14) from subsect. *Alpestres* of sect. *Idaeobatus* was a sister group to the polyploid sect. *Malachobatus*, as well as the polytomy of three sect. *Cyalctis* members. This suggests that some polyploids of *Malachobatus* might originate from common ancestors, via polyploidization of hybrids between *R. pentagonus* and sect. *Cylactis* species. They had experienced species explosion in a short time. Section *Dalibardastrum* species have potential parental lineages from subsects. *Moluccani* and *Stipulosi* of sect. *Malachobatus*. Based on molecular phylogenies, we also provided recommendations for the taxonomic treatments of four taxa. In addition, our results showed certain incongruence between chloroplast and nuclear markers, which might be due to hybridization and introgression.

## Introduction

The genus *Rubus* L. comprises 900–1000 species and has a worldwide distribution (excluding Antarctica) (Focke, 1910, 1911, 1914). It has long been deemed taxonomically challenging due to its complexity for apomixis, polyploidy, lack of a universal species concept, and frequent hybridization (Thompson, 1997). The widely adopted taxonomic system built by Focke (1910, 1911, 1914) divided *Rubus* (*Rubus* hereafter) into 12 subgenera, with the three largest being *Idaeobatus* (117 species), *Malachobatus* (115 species), and *Rubus* (132 species, subg. *Rubus* hereafter). Among the remaining nine subgenera (*Anoplobatus, Chamaebatus, Chamaemorus, Comaropsis, Cylactis*, *Dalibarda, Dalibardastrum, Lampobatus*, and *Orobatus*), only three have more than six species. South-western China is thought to be a major center of origin for *Rubus* because of the rich genetic diversity, large number of subgenera, and extensive morphological variations found there (Lu, 1983). However, the taxonomic system, widely accepted in China, proposed that Chinese *Rubus* consists of eight sections (almost corresponding to the subgenera by Focke), in which 139 species are endemic (Yü et al., 1985; Lu and Boufford, 2003). Some representative sections and the species number are: *Idaeobatus* (88 spp.), *Lampobatus* (1 spp.), *Rubus* (1 spp.), *Malachobatus* (92 spp.), *Dalibardastrum* (11 spp.), *Chamaebatus* (5 spp.), *Cylactis* (9 spp.), and *Chamaemorus* (1 spp.) (Lu and Boufford, 2003). Among these sections, the two largest, *Idaeobatus* and *Malachobatus* are further classified into 11 and 13 subsections respectively (Yü et al., 1985). Both two systems are mainly based on morphological characters. However, the two classification systems are partially contradictory in placement for certain species (Table S1). It is hence a challenging task for researchers to classify *Rubus* species correctly, particularly when just based on morphological appearance.

Polyploidy and hybridization are prevalent in *Rubus* (Alice and Campbell, 1999). Species of sect. *Idaeobatus* are predominantly diploid, whereas sects. *Malachobatus, Dalibardastrum*, and *Chamaebatus* are exclusively polyploid (Thompson, 1997; Naruhashi et al., 2002; Wang et al., 2008). Hybridization in *Rubus* occurs commonly between closely related species (Bammi and Olmo, 1966; Iwatsubo and Naruhashi, 1991, 1992, 1993a, 1996, 1998; Randell et al., 2004; Mimura et al., 2014), but sometimes can occur between sections (Iwatsubo and Naruhashi, 1993b; Alice et al., 2001). For instance, *Rubus parvifolius* of sect. *Idaeobatus* can not only cross with *R. coreanus* (*R*. × *hiraseanus*, 2*x* and 3*x*) and *R. phoenicolasius* (*R*. × *nikaii*, 2*x*) from sect. *Idaeobatus* (Iwatsubo and Naruhashi, 1991, 1996, 1998), but also with *R. sieboldii* (*R*. × *tawadanus*, 3*x*) from sect. *Malachobatus* (Iwatsubo and Naruhashi, 1992, 1993b), resulting in ranges of morphological variations (Nybom and Schaal, 1990). Therefore, molecular data can be extremely useful in assessing the phylogenetic relationship among *Rubus* species to complement with morphological data.

A lot of studies have attempted to gain phylogenetic information within *Rubus* using either maternally inherited chloroplast markers or a bi-parentally inherited ribosomal DNA marker. Molecular data, such as *ndh*F (Howarth et al., 1997; Morden et al., 2003; Zhang et al., 2015), *rbc*L (Imanishi et al., 2008), *rpl*16 (Alice et al., 2008), *trn*G-*trn*S (Michael, 2006), *trn*L-*trn*F (Yang and Pak, 2006), and ITS (Alice and Campbell, 1999; Alice et al., 2001), have partially resolved some phylogenetic uncertainties of *Rubus*. However, most of these studies focused on European and American *Rubus* taxa (Alice and Campbell, 1999; Sochor et al., 2015). The phylogeny of Chinese *Rubus*, with a majority of endemic taxa remains unresolved to date. Research with a more extensive taxon sampling based on multiple chloroplast and nuclear regions is necessary to construct a comprehensive phylogeny within Chinese *Rubus*.

In terms of polyploidy, the use of low-copy nuclear genes (LCNGs) is particularly useful for reconstructing reticulate evolution (Zimmer and Wen, 2013). Previous phylogenetic studies have shown that granule-bound starch synthase I (*GBSS*I) exons and introns are useful in resolving relationships among closely-related species or genera (Rousseau-Gueutin et al., 2009), especially in detecting ancient hybridizations (Evans et al., 2000; Michael, 2006). Phosphoenolpyruvate carboxylase (*PEPC*) has also been reported to have one or few copies and to be phylogenetically informative in different flowering plant families (Lo et al., 2009). These LCNGs have been applied to reconstruct the phylogeny within the Rosaceae family (Evans et al., 2000; Lo et al., 2009; Rousseau-Gueutin et al., 2009), revealing promising prospects. Therefore, we also expected to find informative characters within Chinese *Rubus* by using these low copy nuclear genes.

In this study, we used three chloroplast (*rbc*L, *rpl*20-*rps*12, and *trn*G-*trn*S) and three nuclear (*nrITS, GBSS*I-2, and *PEPC*) genetic markers to reconstruct the phylogeny of Chinese *Rubus*. Our sampling covered 106 species from 17 out of 24 subsections (six out of eight sections) in *Rubus*. There are four specific objectives of this study: (1) to evaluate the phylogenetic information of the six markers at different taxonomic levels; (2) to obtain a well-resolved and thoroughly sampled phylogeny for Chinese *Rubus*; (3) to illustrate the evolutionary history for sects. *Idaeobatus, Malachobatus*, and *Dalibardastrum*; and (4) to provide recommendations for the taxonomic treatments of four taxa based on molecular phylogenies.

## Materials and methods

### Taxon sampling

In total, we sampled 142 *Rubus* individuals, of which 88 (representing 63 species) belong to sect. *Idaeobatus*, one belongs to sect. *Rubus*, 47 (representing 36 species) belong to sect. *Malachobatus*, two belong to sect. *Dalibardastrum*, one belongs to sect. *Chamaebatus*, and three belong to sect. *Cylactis*. This collection, with confirmed ploidy level, contains 70 diploids (2*n* = 2*x* = 14), one triploid (2*n* = 3*x* = 21), 38 tetraploids (2*n* = 4*x* = 28), three hexaploids (2*n* = 6*x* = 42), and one octoploid (2*n* = 8*x* = 56) (Table S1) (Thompson, 1997; Amsellem et al., 2001; Meng and Finn, 2002; Wang et al., 2008). Samples were collected in the wild field from Sichuan, Guizhou, Jiangxi, He'nan, Shaanxi, Gansu, and Tibet Province, China (Figure S1) and were all identified by at least three botanists. Voucher specimens were deposited in the herbarium for horticultural plants, Sichuan Agricultural University (These herbaria were not indexed in Index Herebariorum). The samples were classified according to Flora of China (Yü et al., 1985; Lu and Boufford, 2003) because of the endemicity of some species. *Fragaria vesca* L. and *Rosa banksiae* Ait. were chosen as outgroups based on a previous study (Morgan et al., 1994). Detailed information can be found in Table S1.

### DNA extraction, PCR amplification, and sequencing

Total genomic DNA was isolated from silica-gel dried or frozen leaf tissues using a modified cetyltrimethyl ammonium bromide (CTAB) method (Zhou, 2005). Three chloroplast regions (*rbc*L, *rpl*20-*rps*12, and *trn*G-*trn*S), nuclear ribosomal internal transcribed spacers (*nrITS*), and two single copy nuclear genes coding for granule-bound starch synthase I (*GBSS*I), and phosphoenolpyruvate carboxylase (*PEPC*) were used in this study. The family Rosaceae has two copies of *GBSS*I: *GBSS*I-1 and *GBSS*I-2 (Evans et al., 2000). We selected *GBSS*I-2 gene with primers from strawberry (Rousseau-Gueutin et al., 2009) due to the observations of multiple copies of *GBSS*I-1 in *Rubus* polyploids during preliminary screening. Primers for above markers and the corresponding annealing temperature used in this study are listed in Table 1.

**Table 1:** **Primers for chloroplast and nuclear amplification in this study**.

**Region**	**Primer sequence (5′-3′)**	**Tm (°C)**	**Amplified length (bp)**	**References**
**CHLOROPLAST REGIONS**				
*rbc*L	1F: ATGTCACCACAAACAGAAAC 724R: TCGCATGTACCTGCAGTAGC	55	700	Hasebe et al., 1993
*rpl*20-*rps*12	F: TTTGTTCTACGTCTCCGAGC R: GTCGAGGAACATGTACTAGG	55	800	Hamilton, 1999
*trn*G-*trn*S	F: GAACGAATCACACTTTTACCAC R: GCCGCTTTAGTCCACTCAGC	58	700	Hamilton, 1999
**NUCLEAR REGIONS**				
*nrITS*	ITS5: GGAAGTAAAAGTCGTAACAAGG ITS4: TCCTCCGCTATATGATATGC	55	700	White et al., 1990
*GBSS*I-2	F2: TGGTCTTGGTGATGTTCTTGG R2: GTGTAGTTGGTTGTCCTTGTAATCC	58	530–600	Rousseau-Gueutin et al., 2009
*PEPC*	F: CCGKCTTGCWACACCWGAGCTGGAG R: CCRGGWGCRTACTCGC	58	750	Lo et al., 2009

PCR amplification was performed in a 25 μL volume containing 20 ng of total DNA, 1.2 μL of MgCl_2_ (25 mmol·L^−1^), 1.4 μL of dNTP mix (10 mmol·L^−1^), 1 μL of each primer (5 μmol·L^−1^), 1.5 U of PfuDNA polymerase (Tiangen, Beijing), and 2.0 μL of 10 × PCR buffer (10 mmol·L^−1^ pH 8.0 Tris-HCl, 50 mmol·L^−1^ KCl, 1.5 mmol·L^−1^ EDTA). Conditions for amplification consisted of an initial denaturation at 94°C for 4 min, followed by 35 cycles at 94°C for 45 s, then at 55–58°C for 1 min and at 72°C for 1 min, with a final extension at 72°C for 10 min. Amplifications were conducted using a PTC-200 thermocycler (Bio-rad, Hercules, CA).

All reported genes in this study gave only one single band as determined by 1% agarose gel electrophosis. The products were purified using the UNIQ-10 Column MicroDNA Gel Extraction Kit (Sangon, Shanghai, China). Then they were sequenced directly in both directions using Big Dye Terminator Cycle Sequencing kit (version 2.0, Applied Biosystems, Foster City, CA, USA) on an ABI PRISM 3730 (Applied Biosystems, Foster City, CA, USA) automatic DNA sequencer (Beijing Genomics Institute (BGI), Shenzhen). All the sequences were deposited in the GenBank database with the following accession numbers: KU881049-KU881624, KU891076-KU891200, and KU926720-KU926855 (Table S1).

### Sequence alignment and phylogenetic analyses

Sequences of the six examined regions were edited and assembled using CLC Genomics Workbench (v7.5, CLC bio, Qiagen, Boston, MA). After manually editing, final datasets were aligned separately with Muscle (Edgar, 2004), and adjusted in the Molecular Evolutionary Genetics Analysis software (MEGA6) (Tamura et al., 2013) with gaps treated as missing data.

Before tree reconstruction, we performed partition homogeneity test (PHT) in PAUP v4.0 b10 (1000 replicates, invariable sites excluded) for the plastic and nuclear datasets (Swofford, 2002). According to the obtained PHT results, we applied partitioned phylogeny analyses by using maximum likelihood (ML) and bayesian inference (BI) methods for the combined cpDNA or nDNA datasets, with assignment that each partition had its own evolutionary rate. DNA substitution models were selected out from JModelTest v2.1.1 (Darriba et al., 2012) according to Akaike Information Criterion (AIC) (Akaike, 1974) for each gene.

We inferred the ML trees using the edge-linked partitioned phylogeny in IQ-TREE v1.4.2, which could implemented individual assigned substitution models for each partition (Nguyen et al., 2015; Chernomor et al., 2016). One thousand regular bootstrap replicates were performed to obtain confidence values for the branches. The values were considered to be low when strictly inferior to 65%, moderate between 65 and 80% and strong when superior to 80%. BI analyses were performed using MrBayes v3.2.1 with partitioned genes (Ronquist et al., 2012). The Markov chains Monte Carlo (MCMC) algorithm was run for 6,000,000 generations with one cold and three heated chains, starting with a random tree and sampling one tree every 1000 generations. The first 1,500,000 generations were treated as burn-in. An adequate burn-in value for each analysis was assessed using the software Tracer 1.5 (Rambaut and Drummond, 2009) and a 50% majority-rule consensus tree was then computed.

Phylogenetic network was constructed for combined nuclear datasets using SplitTree v4.14.2 (Huson and Bryant, 2006). Network analysis was performed using the NeighborNet algorithm with Kimura 2-parameter (K2P) distance and Ordinary Least Square Method implemented.

## Results

### Phylogeny of combined chloroplast regions

After treating the gaps as missing characters, our aligned chloroplast *rbc*L, *rpl*20-*rps*12, and *trn*G-*trn*S DNA regions contained 668 base pairs (bp), 771 bp, and 694 bp in length, respectively (Table 2). The final combined cpDNA matrix consisted of 144 taxa and 2133 bp, of which 297 (13.92%) were variable. The selected best fit models for *rbc*L, *rpl*20-*rps*12 and *trn*G-*trn*S were TIM3+I+G, TVM+I+G, and GTR+I+G, respectively.

**Table 2:** **Comparison of sequence variation and best-fitting models among different markers utilized in *Rubus***.

**Region**	***rbc*L**	***rpl*20-*rps*12**	***trn*G-*trn*S**	**Combined cpDNA**	***nrITS***				***GBSS*I-2**	***PEPC***	**Combined nDNA^a^**
					**ITS**	**ITS1**	**5.8S**	**ITS2**			
**NUMBER OF ACCESSIONS**											
*Rubus* plus outgroups	144	144	144	144	144	144	144	144	136	127	144
*Rubus*	142	142	142	142	142	142	142	142	134	126	142
**ALIGNED NUCLEOTIDE LENGTH (bp)**											
*Rubus* plus outgroups	668	771	694	2133	645	265	164	216	585	681	1911
*Rubus*	668	756	680	2104	640	264	164	212	552	681	1873
**VARIABLE SITES (%)**											
*Rubus* plus outgroups	44 (6.59)	107 (13.88)	146 (21.04)	297 (13.92)	206 (31.94)	108	3	95	205 (35.04)	112 (16.45)	523 (27.37)
*Rubus*	34 (5.09)	90 (11.90)	114 (16.76)	238 (11.31)	159 (24.84)	84	2	73	152 (27.53)	90 (13.22)	401 (21.41)
Within sect. *Idaeobatus*	28 (4.19)	79 (10.45)	108 (15.88)	215 (10.22)	130 (20.31)	—	—	—	123 (22.28)	56 (8.22)	309 (16.50)
Within sect. *Malachobatus*	12 (1.80)	11 (1.46)	12 (1.76)	35 (1.66)	47 (7.34)	—	—	—	39 (7.07)	40 (5.87)	126 (6.73)
AIC selected model^b^	TIM3+I+G	TVM+I+G	GTR+I+G	—	TIM2+I+G	—	—	—	TrN+I+G	GTR+G	—
**BASE FREQUENCIES**											
A	0.2761	0.3019	0.3716	—	0.2149	—	—	—	0.2439	0.2764	—
C	0.2002	0.1951	0.1407	—	0.2813	—	—	—	0.1773	0.2039	—
G	0.2323	0.1420	0.1559	—	0.2720	—	—	—	0.2211	0.2022	—
T	0.2914	0.3610	0.3317	—	0.2318	—	—	—	0.3577	0.3175	—
**SUBSTITUTION MODEL (RATE MATRIX)**											
A-C	2.3823	0.4382	1.1752	—	2.2644	—	—	—	1.0000	0.6428	—
A-G	1.4509	0.8962	1.0455	—	5.2715	—	—	—	2.7068	1.5750	—
A-T	1.0000	0.3380	0.5114	—	2.2644	—	—	—	1.0000	0.9628	—
C-G	2.3823	0.1444	0.0644	—	1.0000	—	—	—	1.0000	0.2635	—
C-T	3.0751	0.8962	1.5332	—	12.8740	—	—	—	1.7668	3.1571	—
G-T	1.0000	1.0000	1.0000	—	1.0000	—	—	—	1.0000	1.0000	—
pinvar	0.8120	0.6200	0.4790	—	0.4080	—	—	—	0.2680	—	—
G	0.6860	0.8810	0.8400	—	0.8540	—	—	—	1.1020	0.2010	—

*AIC, Akaike Information Criterion; pinvar, proportion of invariable sites; G, gamma shape*.

a *The failure nuclear sequences for some samples were treated as missing data according to Wiens and Moen (2008)*.

b *Outgroups included*.

**Figure 1:**
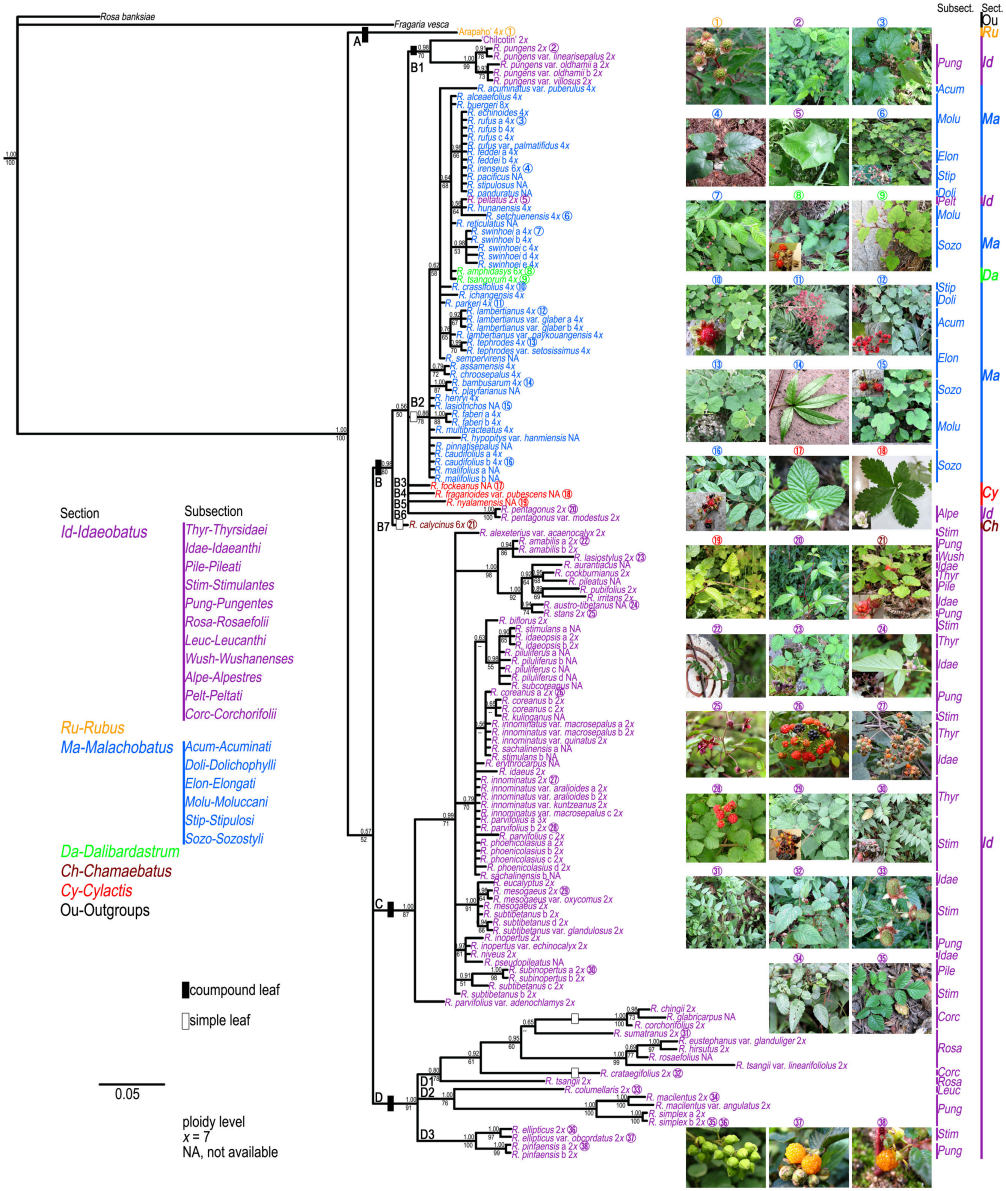
**Bayesian Inference based phylogenetic tree through combined chloroplast datasets**. Posterior probabilities from BI analysis and bootstrap values from ML analysis >50 are provided above and below the branches, respectively. Representative *Rubus* species with typical morphology for each section (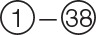) are shown in this figure.

The results of the ML and BI analyses were congruent on major lineages. ML bootstrap support (BS) and BI posterior probabilities (PP) are shown on the 50% majority-rule consensus tree from BI analysis (Figure 1). Four well-supported clades were recovered within Chinese *Rubus*. Clade A included “Arapaho” (Blackberry, *Rubus* spp.), belonging to sect. *Rubus*. Clade B could be divided into seven subclades. Subclade B1 contained “Chilcotin” (Raspberry, *R. idaeus* L.), *R. pungens* and its varieties from subsect. *Pungentes* of sect. *Idaeobatus*. All samples from sect. *Malachobatus* formed a monophyletic subclade (B2) with high support values (78% BS, 0.86 PP). *Rubus tsangorum* and *R. amphidasys* from sect. *Dalibardastrum* were nested within subclade B2. *Rubus peltatus* from subsect. *Peltati* of sect. *Idaeobatus* clustered with two subsect. *Moluccani* species of sect. *Malachobatus* with weak support (64% BS, 0.56 PP). *Rubus fockeanus* (B3), *R. fragarioides* var. *pubescens* (B4), and *R. nyalamensis* (B5) from sect. *Cylactis, R. pentagonus* and *R. pentagonus* var. *modestus* (B6) from subsect. *Alpestres* of sect. *Idaeobatus* were in a polytomy with subclade B2. Subclade B7 was represented by *R. calycinus* of sect. *Chamaebatus* and sister to the group of subclades B1-B6. The above mentioned subclades formed a well-supported (80% BS, 0.98 PP) clade B.

The polyphyly of sect. *Idaeobatus* was strongly supported in the cpDNA tree (Figure 1). Clade C included all samples from subsections *Thyrsidaei, Idaeanthi, Pileati*, and *Wushanenses*, as well as most species from *Stimulantes*, and *Pungentes* of sect. *Idaeobatus*. The phylogenetic relationships among closely related species of clade C were not well-resolved. Clade D was composed of three subclades: a well-supported subclade D1 with samples from subsects. *Rosaefolii* and *Corchorifolii*; D2 with *R. macilentus, R. macilentus* var. *angulatus*, and *R. simplex* of subsect. *Pungentes*, as well as *R. columellaris* of subsect. *Leucanthi*; D3 with *R. ellipticus* and *R. ellipticus* var. *obcordatus* of subsect. *Stimulantes*, and two samples of *R. pinfansis* of subsect. *Pungentes*.

### Phylogeny of nuclear markers

Via carefully checking the original tracing data, we detected few allelic like variations in ITS (3 out of 630 bp), *GBSS*I-2 (6 out of 491 bp), and *PEPC* datasets (2 out of 680 bp). Since each marker only gave single PCR product band, these variations seem more likely to be allelic sites, but might also be the consequences of PCR-generated mutations, which is inevitable with this methodological approach. All these sites that might interfere the accuracy of phylogeny were manually deleted as Brysting et al. (2011) did in their studies. We did not detect any other variations like insertion/deletion events of these three genes among the individuals of same species, even in the polyploids.

The ITS alignment of *Rubus* included 264 bp of ITS1, 164 bp of 5.8S rDNA, 212 bp of ITS2 (Table 2). Together with the outgroups, the final *nrITS* dataset contained 144 accessions and 645 aligned nucleotides, of which 206 (31.94%) were variable.

For the *GBSS*I-2 gene, we failed in getting the sequences for *R. pileatus, R. kulinganus, R. simplex, R. acuminatus* var. *puberulus*. Too much noise was found in the sequencing chromatograms (mostly after polyA segment) of these four taxa, which made it impossible to call out the base accurately, even after several runs of sequencing replication. While for other four samples from subsect. *Corchorifolii*, we only obtained partial sequence of *GBSS*I-1 using the same primers. We excluded these eight species in the following assay. Finally, the *GBSS*I-2 dataset included 136 accessions and 585 aligned nucleotides that contained 205 (35.04%) variable sites.

For *PEPC*, we failed to obtain sequences for 18 samples. Thirteen failed because of no PCR products, and other five samples failed due to too much noise in the tracing data. Among these, there are 16 diploids from subsects. *Pungentes, Rosaefolii, Leucanthi*, and *Corchorifolii* of sect. *Idaeobatus*, one tetraploid of sect. *Malachobatus*, and *Rosa banksiae*. The sequence for *Fragaria vesca* (XM011462481) was obtained from GenBank. Final *PEPC* dataset contained 127 accessions and 681 aligned nucleotides, which included 112 (16.45%) variable sites, much less than that of ITS and *GBSS*I-2 markers.

**Figure 2:**
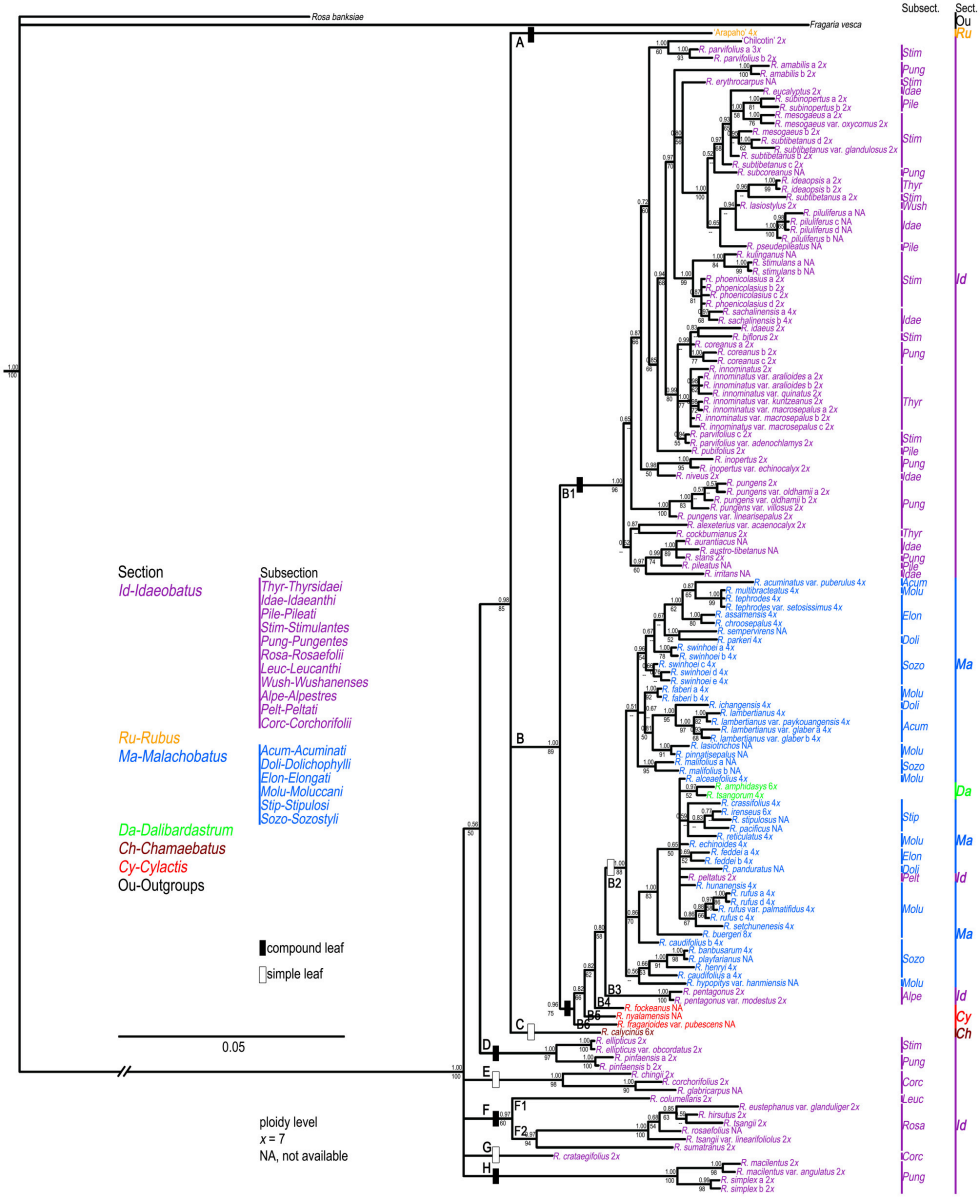
**Bayesian Inference based phylogenetic tree through combined nuclear datasets**. Double slashes on branches indicate branch lengths not in proportion. Posterior probabilities and bootstrap values >50 are provided above and below the branches, respectively.

Based on the theoretic research by Wiens and Moen (2008), we treated those failure sequences as missing data for tree reconstruction when combined nuclear datasets. PHT indicated significant incongruence (*P* = 0.001) among the three nDNA markers. TIM2+I+G, TrN+I+G, and GTR+G models were then selected as best models for them (Table 2) when constructing partitioned phylogeny. Phylogenetic analyses of combined nDNA datasets using ML and BI methods showed similar topologies (Figure 2). Neither sect. *Idaeobatus* nor sect. *Cylactis* was recovered as monophyletic. In addition, those subclades, covering sects. *Malachobatus* plus *Dalibardastrum* and *R. pelataus* (B2), and *Cylactis* (B4–B6), as well as *R. pentagonus* (B3) identified in the cpDNA tree, were also supported here, while sect. *Chamaebatus* formed a separate clade C (Figure 2).

Unlike cpDNA results, cultivar “Chilcotin,” and *R. pungens* and its derived varieties all clustered with most sect. *Idaeobatus* species (B1). These *Rubus* samples were nested in a well-supported (89% BS, 1.00 PP) clade B. The remaining five clades (D-H) included 19 samples of sect. *Idaeobatus* (Figure 2), which corresponded to clade D in the chloroplast tree (Figure 1). Clade D of *R. ellipticus, R. ellipticus* var. *obcordatus*, and *R. pinfaensis* was sister to the group (85% BS, 0.98 PP) composed of clades A, B and C. Subsections *Rosaefolii* (F2) and *Leucanthi* (F1) clustered together in clade F (94% BS, 0.97 PP), while four species of subsect. *Corchorifolii* formed clades E and G. Clade H was represented by four samples of subsect. *Pungentes*. Compared with cpDNA tree, the phylogeny within sects. *Idaeobatus* and *Malachobatus* was much better resolved in the nDNA tree.

### Phylogenetic network

**Figure 3:**
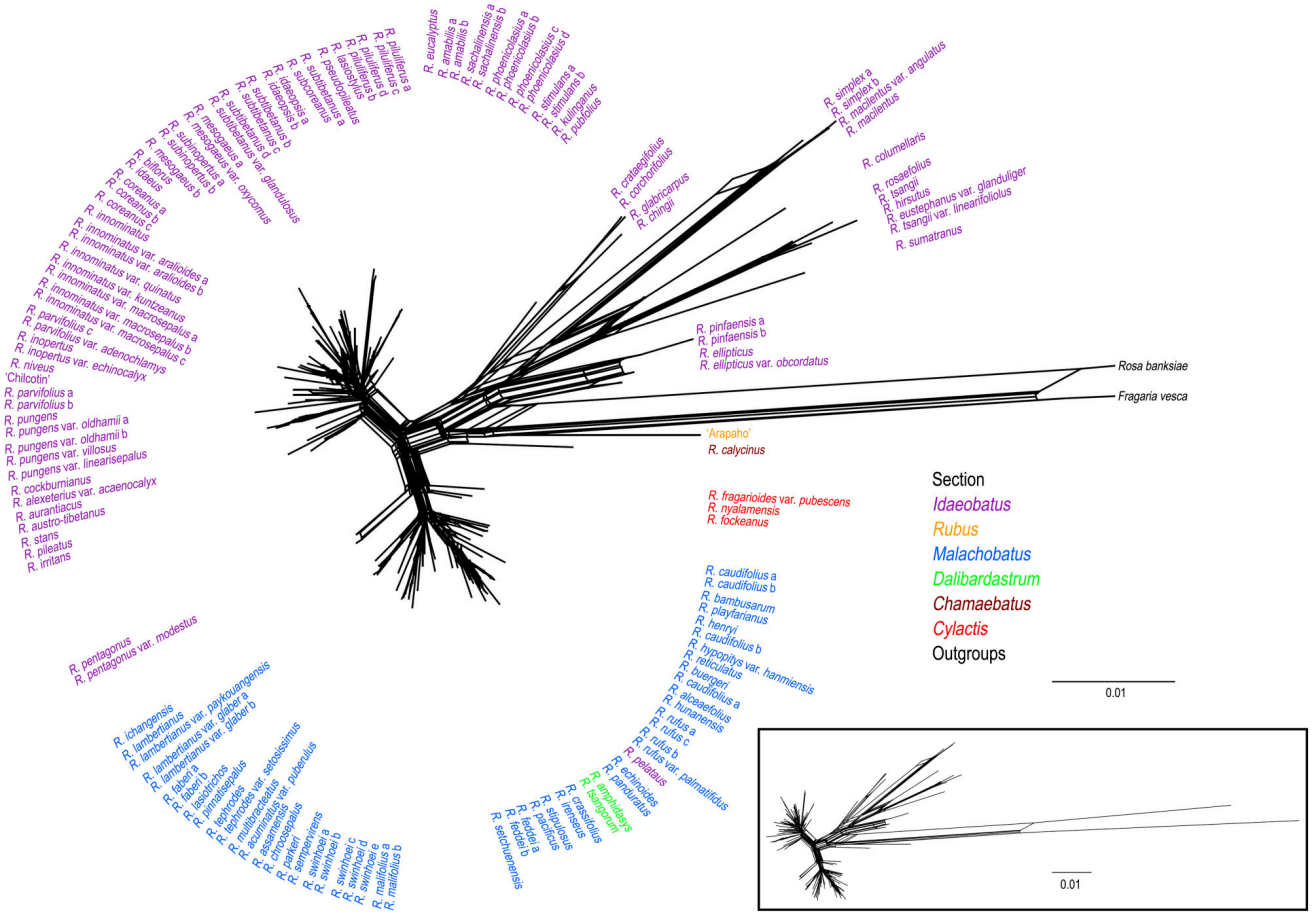
**Phylogenetic network from NeighorNet analysis based on combined nuclear datasets**. Different colors indicate sections in traditional taxonomy.

Phylogenetic network of combined nuclear datasets (Figure 3) was similar to its corresponding phylogenetic tree (Figure 2), which revealed clearer backbones of genus *Rubus*. Section *Idaeobatus* was the most complicated section within *Rubus*, splitting into four distant clades in the network. Species of sect. *Malachobatus* formed a group, which contained *R. tsangorum* and *R. amphidasys* of sect. *Dalibardastrum*, as well as *R. peltatus* of sect. *Idaeobatus*. As predicted, three species of sect. *Cylactis* were split into different clades in nuclear network. Sections *Rubus* and *Chamaebatus* formed separate clades, respectively.

## Discussion

### Sequence variation and phylogenetic incongruence

In this study, *nrITS* and *GBSS*I-2 datasets revealed much higher variation than cpDNA and *PEPC* regions within *Rubus* (Table 2), indicating their promising prospects in the genus. Our results provided insights into the evolutionary history of *Rubus* genus, although the phylogenetic relationships among closely related species were not fully resolved, especially within sects. *Idaeobatus* and *Malachobatus*. Low resolution in phylogeny can be caused by insufficient data, noisy sequences, rapid diversification, polyploidization, and reticulate evolution (Sochor et al., 2015; Spalink et al., 2016). The resolution was limited within sect. *Malachobatus* because of insufficient variation (Table 2). For sect. *Idaeobatus*, some reticulate evolution events were indicated in the nuclear network (Figure 3). When we combined the nuclear datasets (Figure 2), we obtained a much better resolved phylogeny of *Rubus* than each separate marker (Figures S2–4).

Phylogenetic incongruence (within subsects. *Idaeanthi, Stimulantes, Pungentes, Moluccani*, and *Sozostyli* etc.) was detected between the chloroplast and nuclear phylogenies (Figures 1, 2). For example, 19 species within sect. *Idaeobatus* formed a clade (D) in cpDNA tree (Figure 1), while scattered into five clades (D-H) in nDNA tree (Figure 2). *Rubus calycinus* of sect. *Chamaebatus* clustered with groups of sects. *Malachobatus* plus *Dalibardastrum, Cylactis*, as well as *R. pentagonus* in the cpDNA tree (Figure 1), whereas it formed a separate clade (Figure 2). Many studies suggested that convergent evolution, introgression following hybridization, incomplete lineage sorting, and horizontal gene transfer can cause phylogenetic incongruence (Cronn and Wendel, 2004; Linder and Rieseberg, 2004; Zou and Ge, 2008; Wang et al., 2016). Hybridization has occurred in the genus not only between closely related species but also between species from different sections (Bammi and Olmo, 1966; Iwatsubo and Naruhashi, 1991, 1992, 1993a,b, 1996, 1998; Thompson, 1997; Randell et al., 2004; Mimura et al., 2014). Some *Rubus* species were also proved to have undergone hybridization events along with asymmetric introgression (Mimura et al., 2014). Thus, incongruent phylogenetic relationships among our gene trees could be caused by frequent hybridization and genetic introgression.

### Phylogeny of chinese *Rubus*

All our phylogenetic results gave strong support to the monoplyly of genus *Rubus*, consistent with previous studies (Alice and Campbell, 1999; Morden et al., 2003; Yang and Pak, 2006). Based on morphological and chromosomal data, Lu (1983) suggested that evolution in *Rubus* proceeded from woody to herbaceous plants, and from compound to simple leaves. Alice and Campbell (1999) documented that primarily semi-herbaceous, simple-leaved species occupied basal positions in their trees, which was in disagreement with Lu's (1983) hypotheses. Sections *Malachobatus, Dalibardastrum, Chamaebatus*, and *Cylactis* might have common maternal diploid ancestors because they are all close to some sect. *Idaeobatus* species in the chloroplast tree (Figure 1). This also suggested that sect. *Idaeobatus* might be the most primitive group. Then species of different sections experienced distinct evolutionary history, along with various evolutionary rates. Specifically, all these datasets evolved three to six times within sect. *Idaeobatus* than that of sect. *Malachobatus* (Table 2).

#### Section *Idaeobatus*

Section *Idaeobatus* is one of the largest sections in *Rubus*, which contains predominantly diploid species (Thompson, 1997). These analyzed samples occurred in more than four clades within Chinese *Idaeobatus* inferred from different phylogenetic trees (Figures 1, 2). This demonstrated that sect. *Idaeobatus* was a polyphyletic group with at least four independent evolutionary routes. This is congruent with its morphological heterogeneity. Leaf type varies from pinnately compound in species of subsections 1 to 8 (Figure 1, 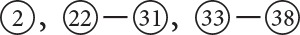), to palmately compound in subsect. *Alpestres* (Figure 1, 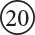), to simple leaves in subsects. *Peltati* and *Corchorifolii* (Figure 1, 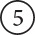, 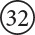).

The first route was the “ancient” group, giving birth to species of subsects. *Thyrsidaei, Idaeanthi, Pileati, Wushanenses*, and a majority of *Stimulantes* and *Pungentes* species (Figures 1, 2). This group is composed of imparipinnately 3-11-foliolate species (Figure 1, 
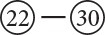
), forming a mixed group without clear circumscription among subsections based on traditional taxonomy. For example, *Rubus aurantiacus, R. austro-tibetanus, R. irritans, R. pileatus*, and *R. stans* from three different subsections formed a well-supported clade (Figures 1, 2). They also share some similar morphological characters (Yü et al., 1985; Lu and Boufford, 2003). It has been documented that these *Idaeobatus* species exhibited both sexual and asexual reproduction, and some species could freely hybridize with each other and could produce fertile hybrids (Iwatsubo and Naruhashi, 1991, 1992, 1993a,b, 1996, 1998; Thompson and Zhao, 1993). This may partly cause their common morphology. Our molecular phylogenies also revealed the complex evolutionary history due to genetic introgression.

As revealed from cpDNA tree (Figure 1), all samples from subsects. *Rosaefolii, Leucanthi, Corchorifolii*, and *R. ellipticus* and *R. ellipticus* var. *obcordatus* from subsect. *Stimulantes*, as well as *R. pinfaensis, R. macilentus, R. macilentus* var. *angulatus*, and *R. simplex* from subsect. *Pungentes* formed a well-supported clade. They not only show notable morphological differences with those species from other subsections, but also reveal high genetic divergence from them. It was evident that a second evolutionary pathway exists within sect. *Idaeobatus*. These species within the pathway are highly similar, with 3-7 leaflets per leaf (except subsect. *Corchorifolii* species with simple leaves, Figure 1, 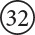), with white flowers in cluster or in corymbs (Figure 1, 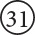
−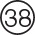). However, these species formed five separate groups in the nDNA tree (Figure 2). The incongruence between chloroplast and nuclear phylogenies indicated that above mentioned species are probably involved in hybridization events. Additionally, subsection *Pungentes* was clearly demonstrated to be polyphyletic in this study. Taxonomic treatments of *R. ellipticus, R. ellipticus* var. *obcordatus*, and *R. pinfaensis* have been fraught with controversy for a long time (Van Thuan, 1968; Lauener, 1970; Yü et al., 1985; Li and He, 2001; Lu and Boufford, 2003; Wang et al., 2009). *Rubus ellipticus* and *R. ellipticus* var. *obcordatus* from subsect. *Stimulantes*, and *R. pinfaensis* from subsect. *Pungentes* formed a well-supported clade, which revealed obvious divergence with any other species from the two subsections by both cpDNA and nDNA trees (Figures 1, 2). Therefore, it seems more reasonable to assign them into another new subsection within sect. *Idaeobatus*.

The third route corresponded to the ancestor of subsect. *Alpestres* (*R. pentagonus*, palmately 3-foliolate leaves, 2*n* = 2*x* = 14) (Figure 1, 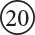), sister to the clade of sect. *Malachobatus* species (Figures 1, 2). As a result, *Rubus pentagonus* and polyploid sect. *Malachobatus* might have a common origin from ancient diploid ancestors.

The fourth pathway produced the subsect. *Peltati* (*R. peltatus*, simple leaves, 2*n* = 2*x* = 14) (Figure 1, 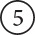), always forming a clade with some species from subsect. *Moluccani* of sect. *Malachobatus* with moderate support. *Rubus peltatus* had common maternal ancestors with *R. setchuenensis* and *R. hunanensis* inferred from cpDNA tree (Figure 1). The species also shares some similar morphology, such as simple leaves and ovate stipules (Figure 1, 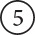), whereas cytogenetic research showed that it is diploid (Thompson and Zhao, 1993). Three suspected diploid species, *Rubus fulvus, R. micropetalus*, and *R. paniculatus*, have been reported to occur in this predominately polyploid section *Malachobatus* (Malik, 1965; Mehra and Dhawan, 1966; Subramanian, 1987). Should *R. peltatus* be moved from subsect. *Peltati* of sect. *Idaeobatus* into subsect. *Moluccani* of sect. *Malachobatus*? It is needed to be taken into consideration more cautiously.

#### Section *Malachobatus*

Section *Malachobatus*, as one of the largest groups in the genus, represents a polyploid complex with tetra-, hexa-, octo- or tetra-decaploids (Thompson, 1997; Naruhashi et al., 2002; Wang et al., 2008). Our samples of this section are all simple-leaved species (Figure 1, 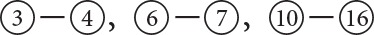). Low variation and limited resolution were obvious within sect. *Malachobaus* (Table 2, Figure 1). A cytogenetic study showed species belonging to six subsections of sect. *Malachobatus* exhibited uniform intra-subsectional karyotypic features (Wang et al., 2008). Low resolution and uniform cytology indicated relatively narrow genetic background among sect. *Malachobatus* species. The taxonomy of subsect. *Moluccani* within *Malachobatus* was problematic (Nybom, 1986). Sixteen samples from this subsection scattered into several clusters in the phylogenetic trees. It seems that the result supported the taxonomy within *Moluccani* by Focke (1910) rather than Yü et al. (1985). Similar results were observed among subsects. *Elongati* and *Sozostyli*.

All samples of sect. *Malachobatus* clustered together in our chloroplast (Figure 1) and nuclear analyses (Figure 2), supporting its monophyly. Interestingly, this group was sister to *R. pentagonus* of sect. *Idaeobatus* and three sect. *Cylactis* members in the cpDNA tree (Figure 1), whereas it formed a polytomy composed of these four species in the nDNA tree (Figures 2). It has been documented that *R. pentagonus* formed a clade with *R. calophyllus* and *R. lineatus* from subsect. *Lineati* of sect. *Malachobatus* (Alice et al., 2008). They also share some morphological similarities, such as unarmed stems, abaxial leaf surfaces, and palmately 3- or 5-foliate leaves (Lu and Boufford, 2003). *Rubus pentagonus* is diploid (2*n* = 2*x* = 14) (Thompson, 1997), while ploidy levels for the three sect. *Cylactis* species have not been reported yet. Moreover, polyploid sect. *Malachobatus* was demonstrated to be of allopolyploid origin (Bammi, 1965; Wang et al., 2015). Therefore, we suggest that some sect. *Malachobatus* polyploids probably originate from common ancestors, via polyploidization of hybrids between *R. pentagonus* and sect. *Cylactis* species.

#### Section *Dalibardastrum*

Section *Dalibardastrum*, represented by *R. tsangorum* and *R. amphidasys*, was nested within sect. *Malachobatus* (Figures 1, 2). This was consistent with a previous study by Alice et al. (2008). The two species share some morphological similarities, such as simple leaves and weak, bristly, prostrate stems (Figure 1, 
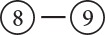
). *Rubus tsangorum* was reported as a tetraploid (2*n* = 4*x* = 28), whereas *R. amphidasys* as a hexaploid (2*n* = 6*x* = 42) (Thompson, 1997). Therefore, it is likely that the two polyploids have at least one parental lineage from sect. *Malachobatus*. Their parental ancestors might be from subsects. *Moluccani* and *Stipulosi* based on phylogenetic trees (Figures 1, 2).

#### Section *Chamaebatus*

In this study, we have collected just one hexaploid species (*R. calycinus*) within sect. *Chamaebatus* (Thompson, 1997), which has simple leaves and sparse prickles or unarmed stems (Figure 1, 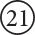). Monophyly of sect. *Chamaebatus* has been demonstrated by Alice and Campbell (1999). *Rubus calycinus* was sister to a large group of sects. *Malachobatus* plus *Dalibardastrum*, and *Cylactis*, as well as *R. pentagenus* of sect. *Idaeobatus* in the chloroplast tree (Figure 1), while it formed a separate clade in the nDNA tree (Figure 2). A possible explanation was that the maternal ancestors of sect. *Idaeobatus* contributed to the formation of *R. calycinus*.

#### Section *Cylactis*

Three examined species of sect. *Cylactis* formed a clearly polyphyletic group (Figures 1, 2). This was also in agreement with its morphological heterogeneity, confirming their different origin patterns by cytological study. Leaf type varies from ternate in *R. fockeanus* (Figure 1, 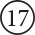), and *R. nyalamensis* (Figure 1, 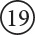), to palmately compound in *R. fragarioides* (Figure 1, 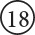), and *R. clivicola*. Stem armature ranges from prickly in *R. fockeanus, R. nyalamensis*, to unarmed in *R. fragarioides*. There are three ploidy levels within this section, diploid (*R. pedatus*), tetraploid (*R. saxatilis*), and mixoploid (*R. arcticus*, 2*x* and 3*x*; *R. humulifolius*, 2*x* and 4*x*) (Thompson, 1997). *Rubus pentagonus* was also demonstrated to be closely related to sect. *Cylactis* (Figures 1, 2). Further research with more samples is needed to illustrate the phylogenetic relationships within sect. *Cylactis*.

### Origin and evolution of polyploids within *Rubus*

Polyploidy has long been considered a major driving force in plant evolution (Soltis and Soltis, 1999). Genus *Rubus* has a large proportion (60%) of polyploidy (Vamosi and Dickinson, 2006), often observed in predominately woody clades (sect. *Malachobatus*). Soltis and Soltis (2009) pointed out that formation of a new species is more likely via allopolyploidy rather than homoploid hybridization. The polyploids within sects. *Malachobatus* and *Chamaemorus* have been demonstrated to be of allopolyploidy origin by Bammi (1965), Michael (2006), and Wang et al. (2015). As predicted, many speciation events in *Rubus* are associated with a change in ploidy levels. Polyploidy in *Rubus* has arisen multiple times, found in subgenera *Rubus, Lampobatus*, and *Malachobatus* (Alice and Campbell, 1999). Multiple origins of polyploid species might also be the main contributor of the morphological complexity in *Rubus* (Rozanova, 1938, see Mavrodiev and Soltis, 2001). Moreover, recurrent hybridization events have been documented occurring over relatively short time spans and geographic distances (Soltis and Soltis, 1999). Ploidy in *Rubus* ranges from diploid (2*n* = 2*x* = 14) to dodecaploid (2*n* = 12*x* = 84), and the most frequent ploidy level is tetraploid (2*n* = 4*x* = 28) (Thompson, 1997). Polyploids show abundant morphological variation, while the genetic background was evident to be narrow in this study. We propose that sect. *Malachobatus* probably experience explosion of species from diploid ancestors to form polyploids within a short time, of which the allotetraploids are the most stable type. The production and fusion of unreduced reproductive cells is likely to be the most predominant pathway leading to allotetraploids (Wang et al., 2010, 2015; Satter et al., 2016).

## Conclusions and perspectives

This study presented phylogenies of genus *Rubus* based on three different kinds of genetic markers (chloroplast DNA, nuclear ribosomal DNA, and single copy nuclear DNA) with a comprehensive taxon sampling. Chloroplast and nuclear markers provided useful information for *Rubus* phylogenies, but general low resolution among closely related species because of insufficient variation and complex evolutionary history. The combined nuclear tree provided the most comprehensive knowledge of the reticulate evolution of *Rubus*. Pervasive incongruence was observed between chloroplast and nuclear trees, which might be due to frequent hybridization and genetic introgression. However, we confirmed that both sects. *Idaeobatus* and *Cylactis* are clearly polyphyletic with species present in several different clades. Section *Malachobatus* was monophyletic, probably originated via polyploidization of hybrids between *R. pentagenus* and sect. *Cylactis* species. Two sect. *Dalibardastrum* species probably have at least one parental lineage from sect. *Malachobatus*.

The molecular phylogenies presented here suggest the need for moderate taxonomic revisions of *Rubus* using modern approaches. Further studies including faster evolving low copy nuclear genes are indispensable and effective to better explore the complex evolutionary history of *Rubus*, especially polyploids. Recently, we have found another low copy nuclear gene, *GBSS*I-1, which showed only one copy in diploids, while exhibited two or three copies in polyploids (unpublished data). It will probably make it possible to obtain a more robust phylogeny, and even illustrate the origin and evolution of the polyploids.

## Author contributions

YW, XW, and HT designed the research. YW carried out the experiments. YW, QC, and TC performed the data analyses. LL contributed to samples collection. YW wrote the manuscript. QC and XW made the revision of the manuscript. All authors approved the final revision to be published.

### Conflict of interest statement

The authors declare that the research was conducted in the absence of any commercial or financial relationships that could be construed as a potential conflict of interest.

## Supplementary material

Figure S1 **Investigation sites of Chinese *Rubus* used for this study**.

Figure S2 **Bayesian Inference based phylogenetic tree through *nrITS* dataset**. Double slashes on branches indicate branch lengths not in proportion. Posterior probabilities and bootstrap values >50 are provided above and below the branches, respectively.

Figure S3 **Bayesian Inference based phylogenetic tree through *GBSS*I-2 dataset**. Double slashes on branches indicate branch lengths not in proportion. Posterior probabilities and bootstrap values >50 are provided above and below the branches, respectively.

Figure S4 **Bayesian Inference based phylogenetic tree through *PEPC* dataset**. Posterior probabilities and bootstrap values >50 are provided above and below the branches, respectively.

Table S1 **List of the studied *Rubus* taxa, their taxonomic position, herbarium voucher information, ploidy level, locality, and the GenBank accession numbers for this study**.

## Abbreviations

BI: bayesian inference
cpDNA: chloroplast DNA
*GBSS*I: granule-bound starch synthase I
ITS: internal transcribed spacer
LCNG: low copy nuclear gene
ML: maximum likelihood
nDNA: nuclear DNA
*PEPC*: phosphoenolpyruvate carboxylase
PHT: partition homogeneity test.
