# Supplementary figures and images for: Phylogenetic Insights into Chinese Rubus (Rosaceae) from Multiple Chloroplast and Nuclear DNAs
Source: Front Plant Sci. 2016 Jun 29;7:968. doi: 10.3389/fpls.2016.00968 (PMC4925702; doi:10.3389/fpls.2016.00968)

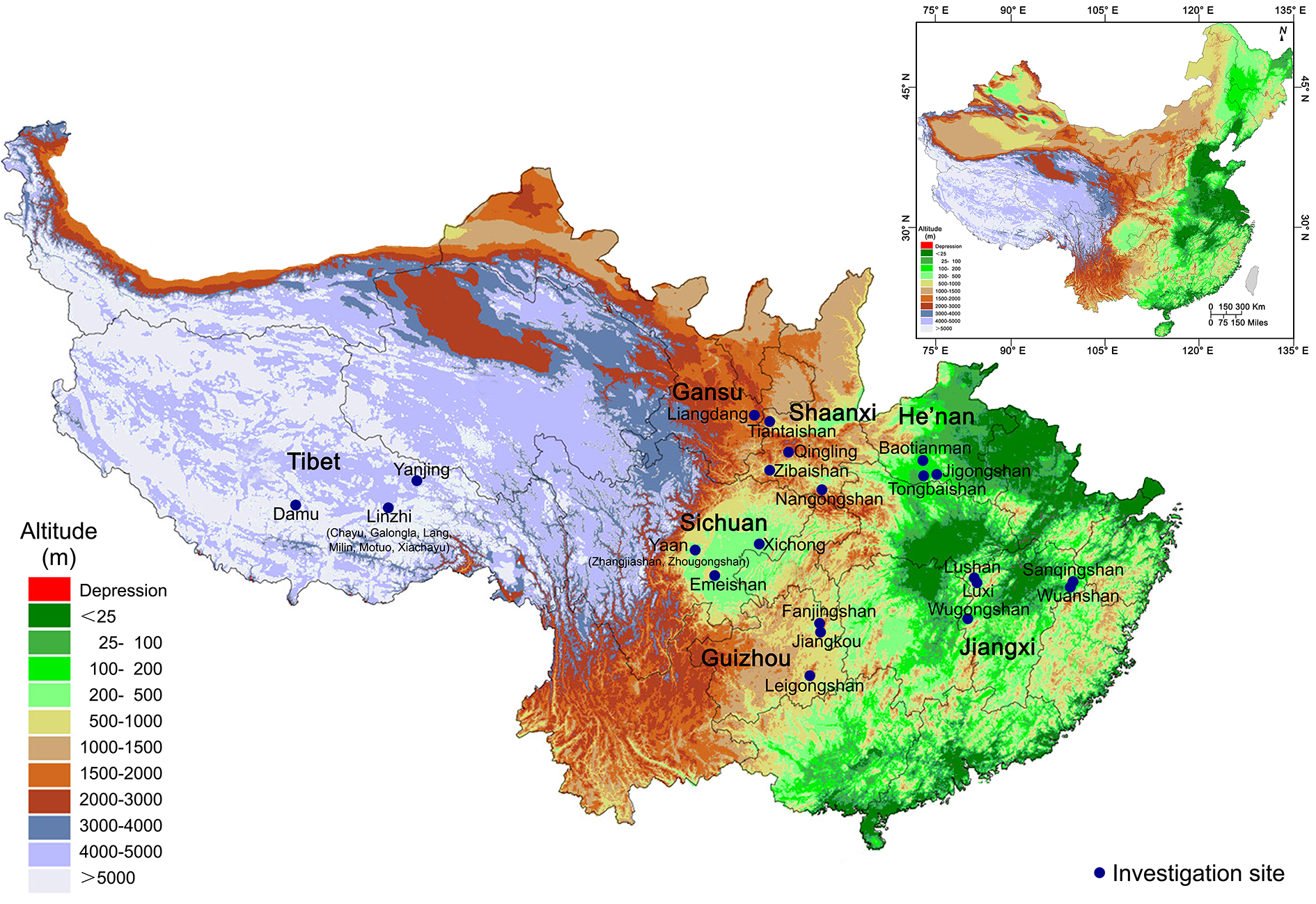

Supplement: Figure S1 — Investigation sites of Chinese Rubus used for this study. [file Image1.tif]

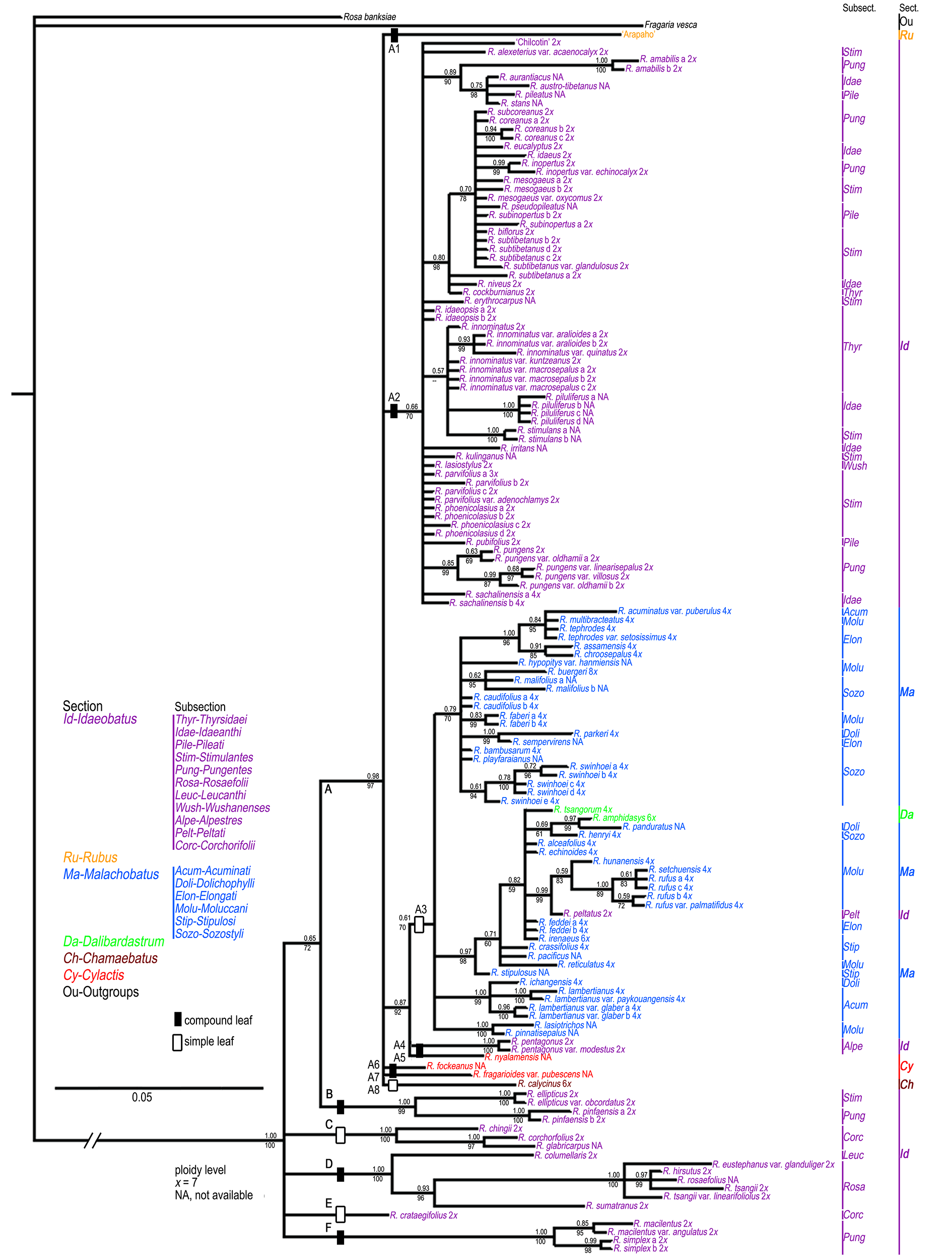

Supplement: Figure S2 — Bayesian Inference based phylogenetic tree through nrITS dataset. Double slashes on branches indicate branch lengths not in proportion. Posterior probabilities and bootstrap values >50 are provided above and below the branches, respectively. [file Image2.tif]

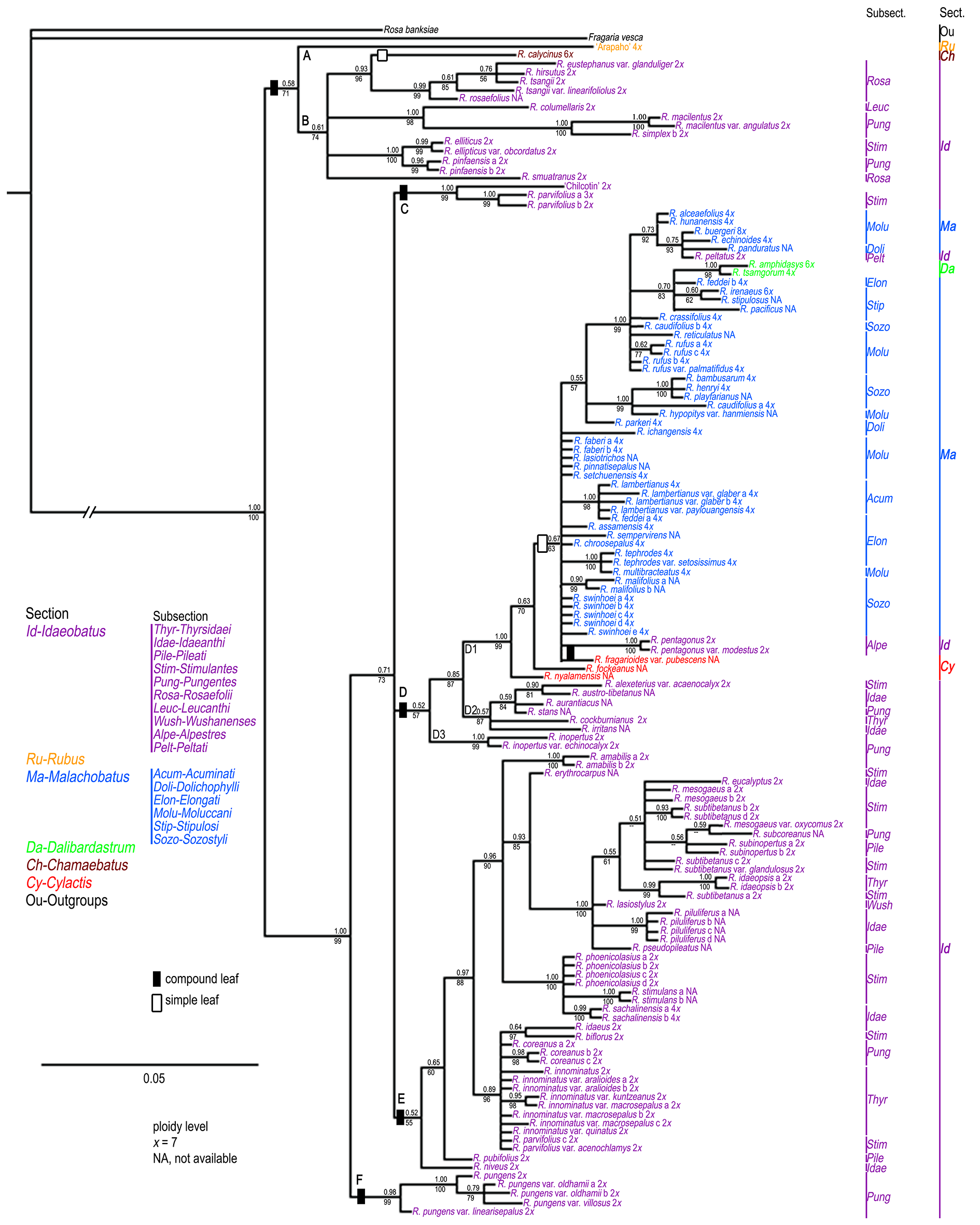

Supplement: Figure S3 — Bayesian Inference based phylogenetic tree through GBSSI-2 dataset. Double slashes on branches indicate branch lengths not in proportion. Posterior probabilities and bootstrap values >50 are provided above and below the branches, respectively. [file Image3.tif]

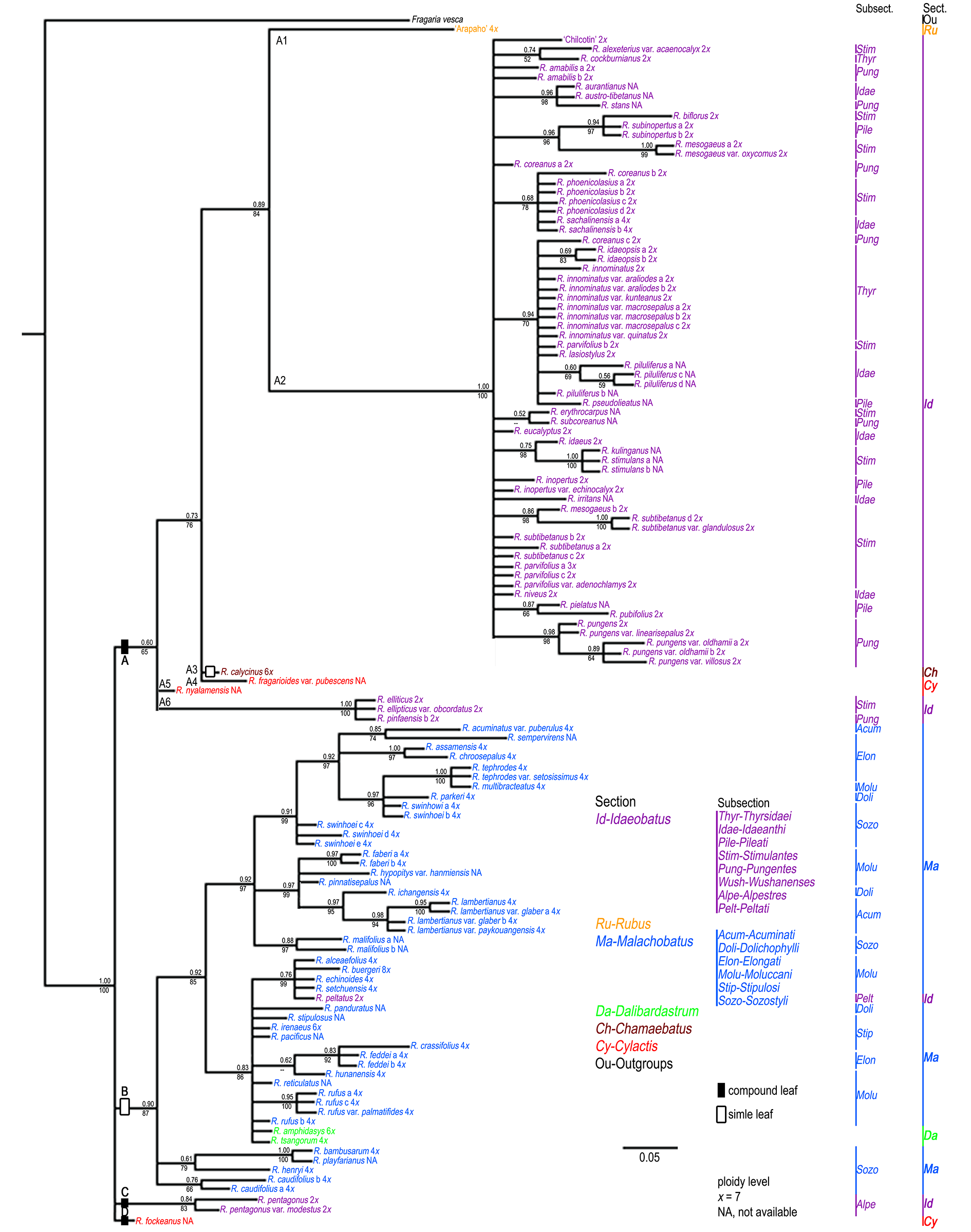

Supplement: Figure S4 — Bayesian Inference based phylogenetic tree through PEPC dataset. Posterior probabilities and bootstrap values >50 are provided above and below the branches, respectively. [file Image4.tif]
